# Influencing factors of children’s physical activity in family

**DOI:** 10.1186/s12889-022-13235-4

**Published:** 2022-04-19

**Authors:** Jie Cai, Yaping Zhao, Jing Wang, Lei Wang

**Affiliations:** 1 School of Physical Education, Shandong University of Finance and Economics, Jinan, China; 2 Library, Shandong Sport University, Jinan, 250000 China; 3 School of Physical Education, Shandong University, Jinan, China; 4 School of Management, Shandong University, Jinan, China

**Keywords:** Children's PA, Social exchange, Perceived benefits, Perceived risks

## Abstract

**Background:**

Children's physical activity (PA) is an important guarantee for children's physical and mental health. Influencing factors of children's PA in family is related to the healthy growth of children and the happy life of families.

**Methods:**

This is a cross-sectional study of influencing factors of children's PA in family. Parents whose children in 15 kindergartens(i.e., children are aged from 3 to 6 years) were sampled.

**Results:**

Government had a significant negative impact on parents’ perceived risks. Community has a significant positive effect on parents’ perceived benefits and a significant negative effect on parents’ perceived risks. Kindergarten has a significant positive effect on parents’ perceived benefits, but has no significant effect on the parents’ perceived risks. Children sports club has a significant positive effect on parents’ perceived benefits. Parents’ perceived benefits has a significant positive impact on children's PA in family, while perceived risks has a significant negative impact.

**Conclusion:**

Government should play a leading role in the development of children's PA in family. Community provides supplementary support. Kindergarten is the key point of developing children's PA in family. Children sports club is the auxiliary force of children's PA in family.

## Introduction

Children's PA is related to children's physical quality and important to the development of a country and a nation. Childhood stage is the key period for the development of human character and emotion, and children's PA is an important guarantee for children's body health and mind health [1]. Children's PA in family refers to the process of cultivating and developing the basic PA ability of children within the family scope [2], more frequent family PA can improved children’s PA [3]. The health problem of children's obesity has become a hot issue in today's society [4], the significance of children's PA has been excavated and re-examined by more and more scholars. However, existing research on children's PA still lacks consideration of parental perception. As the prevalence of childhood overweight and obesity continues to increase, it is essential to unpick how shared family factors impact on children PA [5], but due to the long-term lack of awareness of the importance of children's PA in the China, many parents still believe that children's PA is full of various uncertainties, ignoring the positive effects of children physical activities.

This article based social exchange theory, social exchange theory is also known as exchange theory, first proposed by sociologists Homans [6], points out that people in social activities always want to obtain the biggest benefit with minimal cost, they tend to engage in activities that bring them obvious benefits and rewards. This rational process of balancing gains and losses is called social exchange behavior. Blau [7] proposes that people will estimate their possible benefits in advance in social activities and make comprehensive comparison to select the activities that maximize their benefits. In the process of weighing advantages and disadvantages, people are trying their best to pursue maximum benefits and minimum risks, that is, there are perceived benefits and perceived risks in the process of social exchange behaviour. In terms of social exchange theory, the two most important concepts are perceived benefits and perceived risks, when parents encourage their children to participate in PA, they are bound to comprehensively evaluate what benefits and risks this activity will bring to children [8]. So children's PA in family can also be regarded as a conscious and purposeful social exchange activity. Relevant study shows that children's PA is usually controlled by external factors, and the greatest influence factors are usually parents and other guardians [9]. Other findings showed the levels of teacher and peer’s support also to be important factors in stimulating children's PA [10]. The process of children's PA is an interactive process of individual, material and social environment.

Children's PA in family can not only improve children's physical health level and social development, but also drive family members to participate in PA, which can be regarded as perceived benefits; in exchange, children and their families have to pay costs such as time, energy and money, which can be considered as perceived risks. Therefore, according to social exchange theory, children's PA in families can be regarded as an exchange behavior. This study will take the social exchange theory as the starting point to explore the perceived risks and perceived benefits faced by parents in the decision-making process of whether to support children participating in PA, we hypothesized that government, community, kingdergarten and children sports club influenced parents' perceived benefits and perceived risks, and then affected children's PA in family.

## Materials and methods

### Hypotheses

#### Government and parents’ perceive risks

Government formulates policies and regulations have a huge impact on public awareness and behavior from various perspectives on a macroscopic level, World Health Organization (WHO) regards environmental and policy intervention as the first measure to prevent obesity, government's influence on PA is not only in establishing sports related laws and regulations but also reflected in the other auxiliary policies, etc. Policy and regulation are considered to be the most important factors influencing individual PA [11]. Research shows that the existence of policies does not make people perceive the benefits, but once the absence of policies, people will perceive the existence of risks [12]. The formulated policies are only programmatic provisions, which need to be implemented with the cooperation of the functional departments, otherwise the rights and interests of the public will be damaged, that is, the lack of government functions or ineffective implementation will lead to the loss of rights and interests.

H1: Government will have a negative impact on parents' perceived risk.

#### Community and parents’ perceive risk/benefit

The gradual improvement of community sports facilities has provided good fitness conditions and environment for the public. Community, as an external environment with close relationship between children and their families, can have an important impact on the development of children's PA, and can also alleviate the difficulties of family physical education. Physical education support from community is easier to be accepted by families, and can bring positive influence on family education and children's development with better effect [13]. However, children's outdoor PA space is generally less in China at present, children's outdoor PA facilities are insufficient, the design of community PA environment is unreasonable [14]. All this increases the parents' perception of risk. Parent–child PA is important for children's social and emotional development as well as their future health and well-being, but many parents feel that community-based parent–child physical activities are infrequent and informal [15].

H2a:Community will have a positive impact on parents' perceived benefits.

H2b:Community will have a negative impact on parents' perceived risks.

#### Kingdergarten and parents' perceive risk/benefit

Button [16] found that school sports facilities and equipment will have a positive impact on chindren PA, which is embodied in the sports facilities and equipments' quantity and ease of use. Schools with larger playgrounds and gyms per capita also had higher levels of PA among children [17]. Moreover, Haug et al. [18] found that the amount of students PA with more outdoor activity areas is significantly greater than that of students with less activity areas.Teachers have an important influence on students' participation in PA [19]. Kindergarten teachers play an important role in health promotion, prevention and treatment of childhood obesity [20]. While after the investigation on children's PA in kindergarten in Beijing, it is pointed out that the level of sports equipment in kindergarten has a direct impact on the overall quality of children's PA. On one hand, the physical quality and professional level of preschool teachers will affect the use of equipment function to be able to play, on the other hand, many kindergartens are under the pressure of safety responsibility from parents, as a result, kindergartens are limited in carrying out children's PA [21].

H3a: Kindergarten will have a positive impact on parents' perceived benefits.

H3b: Kindergarten will have a negative impact on parents' perceived risks.

#### Children sports club and parents' perceived benefits

Children sports club mainly provide parent–child sports courses for parents and children or independent sports courses for children, and are committed to improving children's physical skills and comprehensive ability. Children sports clubs are at the meeting point between families and children, which can not only enable children to get physical development, but also benefit children's all-round growth and development [22]. Through Internet platforms sport club obtained accurate family need, use its own resources and characteristics, continue to dig deeply and provide more additional PA services to families, improve the satisfaction of parents and childrens [23].

H4: Children sports club will have a positive impact on parents' perceived benefits.

#### Parents' perceived risks and children's PA in family

Considering safety factors, many parents believe that their children's daily physical activities are enough and there is no need to add extra physical activities. However, parents' misjudgment will have a negative impact on the healthy growth of children over time [24]. Some parents even believe that children sweat after PA easily lead to colds. Some believe that if children spend too much time in PA will lead to no mind to learn or be restless when go to elementary school. Some parents think that children lack safety awareness, PA is prone to risk [25]. Many parents give children a lot of learning courses, such as English, art, thinking training, etc., focus on the development of intelligence, but ignore the children's PA, and even a small number of parents have a psychological resistance to children's PA [26].

H5: Parents' perceived risks will have a negative impact on children's PA in family.

#### Parents' perceived benefits and children's PA in family

The more times children participate in children's PA, the faster their physical quality and skills will improve, and the improvement of physical quality and skills will enhance their self-efficacy [27]. The reason why many parents support their children to participate in PA is that they think it can improve their children's health and help them develop excellent qualities such as self-confidence and courage [28]. Many parents encourage their children to participate in PA in order to improve their physical quality and self-confidence, build a foundation for participating in a variety of social activities, and improve interpersonal skills [29].

H6: Parents' perceived benefits will have a positive impact on children's PA in family.

### Study design

A cross-sectional online survey of parents of Chinese children were conducted. The study was approved by the local Ethics Committee (2021/001, January 17, 2021) and was conducted in accordance with the Declaration of Helsinki as established by the World Medical Association. Completion and submission of the online survey implied consent to participate in this study, which was declared to respondents at the commencement of the survey.

### Procedure

The program was designed by children’s PA experts, pre-school staff, an exercise physiologist and youth exercise coaches. The main challenge is to ensure that the program is widely applicable to other parts of China. Parents volunteered and 350 were randomly selected by computer. In China, kindergartens are seperated from school system, with children aged from 3 to 6 years. Parents attended an all day seminar in which they were acquainted with the program, and were trained by the study team so that they could familiarize with the program. During the program, parents were invited to two additional training days. The goal of these meetings was to collect feed-back on the program. Adherence to the program was followed weekly by the study coordinator and an professional youth coach. At last, 27 parents dropped out of the program because they were busy at work or otherwise, and 323 parents participated in the questionnaire.

Likert seven-grade scale was used for the questionnaire. In order to ensure the content validity of the scale, the scale design was derived from existing literature. On this basis, after several research group discussions and expert opinions, the preliminary design was formed. Then, the parents and kingdergarten teachers were interviewed, according to their feedback, the questionnaire was adjusted and the final questionnaire was determined.

Data collection took place between 1 February and 15 April 2021. Due to the COVID-19pandemic, parents were invited to complete an online questionnaire that was administered via a free online Chinese survey platform (https://www.wjx.cn). To ensure that parents can carefully complete the questionnaire, the one who completed the online questionnaire would get 5 yuan as a reward. At last, 323 were recovered with a recovery rate of 96.3%. Exclude the questionnaire with too fast response speed and regular distribution of answers, 286 were valid, with an effective rate of 88.5%. The ratio of sample amount to item was 13.6:1. The total amount of sample in this study has reached the optimal requirement of structural equation modeling [30]. In addition to the basic information of the participants, the questionnaire involved 7 latent variables, including government, community, kindergarten, children sports club, perceived risks, perceived benefits, and children's PA in family, with a total of 21 items (shown in Table 2). Measurements were performed by the trained technicians who did not know which kindergarten the parents belonged to.

## Results

### Parcitipant characteristics

A total of 286 participants completed the survey (shown in Table 1). Participants were aged between 22 and 40 years (M = 30.5). Among these participants, most were female (62.6%), and Higher than bachelor’s degree(44.8) had Income sufficient for expenditurea(49.0%).

**Table 1:** Demographic characteristics of participants (*n* = 286)

Category	Mean	*n*	%
**Age (years)**	30.5		
**Gender**			
Female		179	62.6
Male		107	37.4
**Education level**			
Less than high school		48	16.8
TAFE/College		110	38.5
Higher than bachelor’s degree		128	44.8
**Family income**			
Income insufficient for expenditure		51	17.8
Balance between income and expenditure		95	33.2
Income sufficient for expenditure		140	49.0

### Reliability and validity test

The reliability and validity of the questionnaire were measured by SPSS 22.0 software. KMO ( Kaiser–Meyer–Olkin) was 0.898, Bartlett’s test of sphericity was 11,042.830, and statistical significance was taken at *p* < 0.05. Cronbach's α coefficient of quesionnaire was 0.936. This means the reliability and validity of the questionnaire had passed the test. Cronbach's α coefficient of all latent variables exceeded 0.7, which met the requirements for internal consistency of the scale, reflecting a good reliability level (Table 2). Using Mplus 7.0 software for further reliability and validity analyses, squared multiple correlations (SMC) values of all items in the questionnaire were greater than 0.36, indicating high reliability of the items (Table 2). The composite reliability (CR) values all exceeded 0.7, which indicates that the reliability of items composition was high. Average variance extracted (AVE) values were all greater than 0.5, which demonstrates that the scale has good convergent validity. Table 3 shows the discriminant validity test results of each latent variable.

**Table 2:** Variables and measurement indicators

Latent variable	Item	Item source	Estimate	SMC	CR	AVE	Cronbach’s α
goverment(gov)	children's PA needs goverment support	Dowda M et al. [31] (2004);	0.918	0.843	0.930	0.816	0.930
	implement policies on children's PA in place		0.835	0.697			
	the content of children's PA is standardized		0.953	0.908			
community (com)	there are sports facilities for children in the community	Welk et al. [32] (2013)	0.851	0.724	0.884	0.718	0.901
	the community regularly carries out children's PA		0.873	0.762			
	the community has enough places for children to play		0.818	0.669			
kingdergarten(gar)	kindergarten organize many physical activities	Button B et al. [16] (2013)	0.880	0.774	0.892	0.734	0.811
	the sports facilities can meet chindren’ needs		0.882	0.778			
	physical education curriculum is reasonable		0.806	0.650			
children sports club(clu)	advanced physical educational concept	Zahner L et al. [22] (2009)	0.761	0.579	0.838	0.633	0.757
	lote of sports fields and equipments		0.836	0.699			
	professional physical education teacher		0.788	0.621			
perceived risks (ris)	safety risks	Keane et al. [26] (2012)	0.655	0.429	0.781	0.547	0.790
	unprofessional children sports equipment may not safe		0.843	0.711			
	children can catch a cold after sweating		0.707	0.500			
perceived benefits (ben)	children become more powerful	Dan et al. [20] (2013)	0.691	0.477	0.877	0.708	0.746
	improve children's balance and coordination		0.887	0.787			
	children's movements become more alert		0.927	0.859			
children's PA in family (fam)	family memnbers often play sports together	Thorn [33] (2008)	0.849	0.721	0.861	0.674	0.840
	children love PA		0.835	0.697			
	encourage and accompany children in PA		0.777	0.604

**Table 3:** Test form for discriminant validity of variables

	AVE	gov	gar	com	clu	ris	ben	fam
gov	0.816	**0.903**						
gar	0.734	0.058	**0.857**					
com	0.718	0.030	0.515	**0.847**				
clu	0.633	0.036	0.264	0.239	**0.796**			
ris	0.547	-0.142	-0.149	-0.127	-0.040	**0.740**		
ben	0.708	0.018	0.539	0.581	0.434	-0.099	**0.841**	
fam	0.674	0.017	0.175	0.186	0.134	-0.113	0.310	**0.823**

The square root of the AVE value of most latent variables was greater than the absolute value of the correlation coefficient between these variables and other latent variables, reflecting higher discriminant validity among the variables incorporated for analyses [34].

### Hypothesis testing

After several revisions, the final analysis model results are shown in Table 4. The fit indices suggest that the model fits the data well. Seven hypotheses were supported and one hypotheses were not. The empirical results and hypotheses are shown in Table 4.

**Table 4:** Parameter estimation and hypothesis testing of analytical models

Path	Estimated value	*P*-Value	Hypothesis
perceived risks ← goverment	0.066	*	H1 surpported
perceived benefits ← community	0.059	***	H2a surpported
perceived risks ← community	0.083	*	H2b surpported
perceived benefits ← kingdergarten	0.061	***	H3a surpported
perceived risks ← kingdergarten	0.083	0.227	H3b not surpported
perceived benefits ← children sports club	0.054	***	H4 surpported
children's PA in family ← perceived risks	0.060	**	H5 surpported
children's PA in family ← perceived benefits	0.049	***	H6 surpported
Fit Index	χ2/df = 350.753/237 = 1.480; RMSEA = 0.050, SRMR = 0.074;CFI = 0.972, TLI = 0.967		

Notes: * *P* < 0.05,** *P *< 0.01,*** *P* < 0.001

The fit indexex of structural equation model in Table 4 are: χ2/ DF = 350.753/237 = 1.480, which meets the requirement of less than 3. RMSEA = 0.050, SRMR = 0.074, meeting the requirements of less than 0.08; CFI = 0.972 and TLI = 0.967 meet the requirement of greater than 0.9. Therefore, all data fit indexes in this paper meet the test standard, indicating that the model matrix is close to the sample matrix and the model is acceptable.

As is showen in Table 4, the path coefficient of government to perceived risks is -0.143 and significant at the level of 0.05, indicating that government has a significant negative effect on perceived risks, H1 is surpported. The path coefficient of community to perceived benefits is 0.379 and significant at the level of 0.001, indicating that community has a significant positive effect on perceived benefits, H2a is surpported. The path coefficient of kindergarten to perceived benefits is 0.280 and significant at the level of 0.001, indicating that kindergarten has a significant positive effect on perceived benefits, H3a is surpported, the path coefficient of kindergarten to perceived risks is not valid, and H3b is not surpported. The path coefficient of children sports club to perceived benefti is 0.266 and significant at the level of 0.001, indicating that children sports club has a significant positive effect on perceived benefits, H4 is surpported. The path coefficient of perceived risks to Children's PA in family is -0.205 and significant at the level of 0.01, indicating that perceived risks has a significant negative effect on children's PA in family, H5 is surpported. The path coefficient of perceived benefits to children's PA in family is 0.564 and significant at the level of 0.001, indicating that perceived benefits has a significant positive effect on children's PA in family, H6 is surpported.

## Discussion

Our results confirm the previously reported positive association between government, community, kindergartens and children sports clubs and children's PA in family [16, 31–33]. In our study, most Chinese kindergarten children met physical activity guidelines of World Health Organisation (WHO) Guidelines on Physical Activity, Sedentary Behaviour and Sleep for Children under 5 Years of Age, verified the previous studies of Chinese scholars [35]. Further, the results of this study substantiate the previous findings that famliy PA is of particular importance regarding children’s PA [20, 26, 35].

The interesting and new finding observed in this study was that in addition to the benefits of physical activity, parents can also feel the possible risks and we are not aware of other previous studies using structural equation modeling in this context that show parents' perceived benefits and risks in children's PA.

Firstly, government had a significant negative effect on perceived risks, indicating that the more and the better government does, the less the parents perceived risks. Environmental contributed a lot to the children in the kindergarten years, while construction of the environment cannot be separated from the guidance of the government [36]. Moderate-to-vigorous physical activity (MVPA) for children varies by policy/practice and the overall quality of kindergartens [31], so parents hope to get more support from the government, such as clear guidance on children's PA from laws, regulations, and policies, which can effectively reduce parents' perceived risks, improve the motivation for children to participate in children's PA, and enhance parents' confidence and security. Government should set up a national example against the sedentary lifestyle of kindergarten children, which did not require major time or financial investment [37].

Secondly, community had a significant positive effect on parents' perceived benefits, and a significant negative effect on parents' perceived risks. It showed that the greater the efforts of the community in children's PA, the greater the perceived benefits of parents, and the smaller the corresponding perceived risks. Parents hope that children can get the opportunity of PA within the community. For example, parents can not only take their children to play games independently in the community, but also hope to have the opportunity to participate in parent–child activities or parent–child sports meetings organized by the community, but the premise is to ensure the safety of children. Most children enjoy an active lifestyle in a community where they can be with friends, participate in diverse activities, experience fun, and increase opportunities for outdoor activities, but they may face time constraints from their parents [38]. This requires more community sport facilities and equipments, community children sport services, community children sports security and so on.

Thirdly, kindergarten has a significant positive effect on parents' perceived benefits, but no significant effect on parents' perceived risks. It shows that the better the kindergarten physical education work is carried out, the greater the parents' perceived benefits will be. Interventions to promote physical activity in preschoolers should focus on kindergartensand encourage involvement of their families [38]. At present, parents have no obvious risk perception for kindergarten physical education. Kindergarten teachers are key players in the success of such a program, their attitude and longterm adherence toward the program should be further studied [37]. Many parents think that kindergarten for children's bounden responsibility, thus PA involved in kindergarten teachers, venues equipment, and course put forward higher request [39], it is gratifying that most parents generally affirm the safety of kindergarten physical education and do not worry about the risk of the development of children physical education.

Fourthly, children sports club has a significant positive effect on parents' perceived benefits, indicating that parents hope to take their children to participate in physical activities in children sports club. children sports club not only provide parents with opportunities for parent–child physical activities, but also allow children to do physical activities in the company of children of the same age. Sports club participation could increase physical activity and hence fitness and to reduce the risk for overweight [33], a range of important social learning, enculturation, and the development of identity arises from participation in the practices of the club [23]. In this case, children's PA will be better carried out in family, children and parents will get more benefits.

Fifthly, parents' perceived risks has a significant negative effect on children's PA in family, and parents' perceived benefits has a significant positive effect on children's PA in family, indicating that children's PA can improve their physical fitness, improve their balance, coordination, sensitivity and other benefits [40], and have a positive effect on whether parents support children to carry out physical activities. But if the relevant safety measures or other factors are not considered, it will also have a negative effect on parents' support for children's PA. It is essential to unpick how shared family factors impact on child weight, female gender, oneparent family type, lower maternal education, lower household class and a heavier parent weight status significantly increased the odds of childhood obesity [26]. Internet driven activities in family increased children sedentary hours [38]. Many parents have a deep perception of the benefits of children's PA in family that can bring physical and mental health development [41], but at the same time, there are still concerns about whether sports are safe or even delay children's learning of other subjects. The parental BMI and education had direct and important influence on children's PA, families with overweight and poorly educated parents need to provide evidence-based health promotion interventions [29].

## Conclusion

The development of children's PA in family needs society support. Government, community, kindergartens and children sports clubs should cooperate to build a social support system for children's PA in family. According to the above analysis, it is believed that the government plays a guiding role in the development of children's PA in family through formulating policies, implementing policies and improving policies. Community provides supplementary support for the development of children's PA in family. Kindergarten is not only the focus of developing children's PA in family, but also play an important role in promoting children physical education to parents. The existence of children sports club plays an auxiliary role in the development of children's PA in family.

## Data Availability

The datasets used and analysed during the current study are available from the corresponding author on reasonable request.

## References

[1] Trost SG, Fees B, Dzewaltowski D (2008). Feasibility and efficacy of a ‘move and learn’ physical activity curriculum in preschool children. J Phys Act Health.

[2] Quarmby T, Dagkas S (2010). Children’s engagement in leisure time physical activity: exploring family structure as a determinant. Leis Stud.

[3] Pluta, B. , Małgorzata Bronikowska, Tomczak, M. , Ida Laudańska-Krzemińska, Michał Bronikowski. Family leisure-time physical activities – results of the "juniors for seniors" 15-week intervention programme. Biomed Human Kinetics. 2017; 9(1).

[4] Robertson W, Friede T, Blissett J, Rudolf M, Wallis M, Stewart-Brown S. Pilot of ’families for health’: community-based family intervention for obesity. Arch Dis Child. 2008;93(11):921–6. 10.1136/adc.2008.139162 18463121

[5] Shrewsbury V, Wardle J (2008). Socioeconomic status and adiposity in childhood: A Systematic Review of Cross-sectional Studies 1990–2005. Obesity.

[6] Homans GC (1958). Social behavior as exchange. Am J Soc.

[7] Blau PM. Exchange and power in social life. Wiley. 1964. https://www.researchgate.net/publication/275714436_Exchange_and_Power_In_Social_Life.

[8] Fiona, Mitchell. Balancing benefit and risk in youth sports. Lancet. Child & adolescent health.2018.

[9] Ryan, RM, Deci, EL Promoting self-determined school engagement: Motivation, learning, and well-being. In K. R. Wenzel & A. Wigfield (Eds.), Educational psychology handbook series. Handbook of motivation at school .2009; p. 171–195.Routledge/Taylor & Francis Group. https://psycnet.apa.org/record/2009- 24219–009.

[10] Michal, B, Malgorzata, B, Ida, LK, Adam, K, Besnik, M, Shemsedin, V. Pe teacher and classmate support in level of physical activity: the role of sex and bmi status in adolescents from kosovo. Biomed Res Int. 2015; 290349. 10.1155/2015/290349 PMC4561870 26380268

[11] Cook, H. , Kohl, H. . Educating the student body: taking physical activity and physical education to school. National Academies Press. 2013; 500 Fifth Street NW, Washington, DC 20001. 24851299

[12] Yli-Piipari S, Watt A, Jaakkola T, Liukkonen J, Nurmi JE (2009). Relationships between physical education students’ motivational profiles, enjoyment, state anxiety, and self-reported physical activity. J Sports Sci Med.

[13] McKeown, Kieran. Strategic framework for family support within the family and community services resource center programme. Fam Supp Agency. 2013; 6:84.

[14] Granado-Villar DC, Gitterman BA, Brown JM, Chilton LA, Zind B (2013). Community pediatrics: navigating the intersection of medicine, public health, and social determinants of children’s health. Pediatrics.

[15] O'Connor, A, Skouteris, H, Nolan, A, Hooley, M, Cann, W, Williams-Smith, J. Applying intervention mapping to develop an early childhood educators' intervention promoting parent–child relationships. Early Child Dev Care. 2017;1–18. 10.1080/03004430.2017.1362401.

[16] Button B, Trites S, Janssen I (2013). Relations between the school physical environment and school social capital with student physical activity levels. BMC Public Health.

[17] Sara, D'Haese, Delfien, Van, Dyck, Ilse, et al.. Effectiveness and feasibility of lowering playground density during recess to promote physical activity and decrease sedentary time at primary school. BMC Public Health. 2013;13(1),1154–1163. 10.1186/1471-2458-13-1154. 10.1186/1471-2458-13-1154 PMC3878886 24325655

[18] Haug E, Torsheim T, Sallis JF (2008). The characteristics of the outdoor school environment associated with physical activity. Health Educ Res.

[19] Bergh IH, Bjelland M, Grydeland M, Lien N, Andersen LF, Klepp KI (2012). Mid-way and post-intervention effects on potential determinants of physical activity and sedentary behavior, results of the heia study - a multi-component school-based randomized trial. Int J Behav Nutr Phys Activity.

[20] Dan, N, Geva, D, Pantanowitz, M, Igbaria, N, Eliakim, A. Long term effects of a health promotion intervention in low socioeconomic arab-israeli kindergartens. Bmc Pediatr, 2013; 13. 10.1186/1471-2431-13-45 PMC3648431 23547765

[21] Tucker P (2008). The physical activity levels of preschool-aged children: a systematic review. Early Child Res Q.

[22] Zahner L, Muehlbauer T, Schmid M (2009). Association of sports club participation with fitness and fatness in children. Med Sci Sports Exerc.

[23] Light RL (2010). Children’s social and personal development through sport: a case study of an australian swimming club. J Sport Soc Issues.

[24] Bentley GF, Goodred JK, Russell J, Sebire SJ, Lucas PJ, Fox KR (2012). Parents’ views on child physical activity and their implications for physical activity parenting interventions: a qualitative study. BMC Pediatr.

[25] Ulf, Ekelund, Jian'an, Luan, Lauren, B, et al. Moderate to vigorous physical activity and sedentary time and cardiometabolic risk factors in children and adolescents. JAMA. 2012,307(7),704–712. 10.1001/jama.2012.156. 10.1001/jama.2012.156 PMC3793121 22337681

[26] Keane E , Layte R , Harrington J , et al. Measured parental weight status and familial socio-economic status correlates with childhood overweight and obesity at age 9. PLoS ONE. 2012; 7. 10.1371/journal.pone.0043503 PMC3422292 22912886

[27] Trost SG, Sallis JF, Pate RR, Freedson PS, Taylor WC, Dowda M (2003). Evaluating a model of parental influence on youth physical activity. Am J Prev Med.

[28] Lee H, Tamminen KA, Clark AM, Slater L, Spence JC, Holt NL (2015). A meta-study of qualitative research examining determinants of children’s independent active free play. Int J Behav Nutr Phys Act.

[29] Parikka S , M?Ki P , Lev?Lahti E , et al. Associations between parental BMI, socioeconomic factors, family structure and overweight in Finnish children: a path model approach. BMC Public Health, 2015; 15(1):271. 10.1186/s12889-015-1548-1 PMC4371876 25885334

[30] Sharma S, Crossler RE (2014). Disclosing too much? situational factors affecting information disclosure in social commerce environment. Electron Commer Res Appl.

[31] Dowda M, Pate RR, Trost SG (2004). Influences of preschool policies and practices on children’s physical activity. J Community Health.

[32] Welk, G. J. , Corbin, C. B. , D Dale. Measurement issues in the assessment of physical activity in children. Research Quarterly for Exercise and Sport. 2000;71(sup2), 59–73. 10.1080/02701367.2000.11082788. 10.1080/02701367.2000.11082788 25680015

[33] Thorn JE, Delellis N, Chandler JP, Boyd K (2013). Parent and child self-reports of dietary behaviors, physical activity, andscreen time. J Pediatr.

[34] Fornell C, Larcker DF (1981). Structural equation models with unobservable variables and measurement error: algebra and statistics. J Mark Res.

[35] Guan H, Zhang Z, Wang B (2020). Proportion of kindergarten children meeting the WHO guidelines on physical activity, sedentary behaviour and sleep and associations with adiposity in urban Beijing. BMC Pediatr.

[36] Nemet D, Geva D, Eliakim A (2011). Health promotion intervention in low socioeconomic kindergarten children. J Pediatr.

[37] Sigmundová, Dagmar, Sigmund E , Badura P , et al. Weekday-weekend patterns of physical activity and screen time in parents and their pre-schoolers. Bmc Public Health. 2016; 16(1):898. 10.1186/s12889-016-3586-8 PMC5004262 27576897

[38] D Tannehill, Macphail A , Walsh J , et al. What young people say about physical activity: the children's sport participation and physical activity (CSPPA) study[J]. Sport Education & Society. 2015; 20(4):442–462.

[39] Pamela Hodges Kulinna. Models for curriculum and pedagogy in elementary school physical education. Elementary School J. 2008; 108:3, 219–227

[40] Reilly JJ (2010). Low levels of objectively measured physical activity in preschoolers in childcare. Med Sci Sports Exerc.

[41] Reilly JJ, McDowell ZC (2003). Physical activity interventions in the prevention and treatment of paediatric obesity: systematic review and critical appraisal. Proc Nutr Soc.

